# Moku virus; a new *Iflavirus* found in wasps, honey bees and Varroa

**DOI:** 10.1038/srep34983

**Published:** 2016-10-07

**Authors:** Gideon J Mordecai, Laura E Brettell, Purnima Pachori, Ethel M. Villalobos, Stephen J Martin, Ian M Jones, Declan C Schroeder

**Affiliations:** 1 Viral Ecology, Marine Biological Association, Plymouth PL7 5BU, UK; 2 School of Biological Sciences, University of Reading, Reading RG6 6AJ, UK; 3 School of Environment and Life Sciences, University of Salford, Manchester M5 4WT, UK; 4 The Genome Analysis Centre, Norwich Research Park, Norwich NR4 7UH, UK; 5 Department of Plant and Environmental Protection Sciences, University of Hawaii, Manoa, HI 96822, USA.

## Abstract

There is an increasing global trend of emerging infectious diseases (EIDs) affecting a wide range of species, including honey bees. The global epidemic of the single stranded RNA Deformed wing virus (DWV), driven by the spread of *Varroa destructor* has been well documented. However, DWV is just one of many insect RNA viruses which infect a wide range of hosts. Here we report the full genome sequence of a novel *Iflavirus* named Moku virus (MV), discovered in the social wasp *Vespula pensylvanica* collected in Hawaii. The novel genome is 10,056 nucleotides long and encodes a polyprotein of 3050 amino acids. Phylogenetic analysis showed that MV is most closely related to Slow bee paralysis virus (SBPV), which is highly virulent in honey bees but rarely detected. Worryingly, MV sequences were also detected in honey bees and Varroa from the same location, suggesting that MV can also infect other hymenopteran and Acari hosts.

Emerging and re-emerging diseases affecting a diverse range of organisms pose an ongoing threat to global health and food security. The transmission of DWV around the world in conjunction with Varroa is a well-studied example of an emerging insect pathogen^1^,^2^. The spread of Varroa from Asia to the rest of the world was mirrored by the spread of DWV throughout the European bee populations^2^ and introduced a new transmission route for the virus, leading to selection of a virulent strain, DWV type A, which replicates to high levels and results in colony collapse^1^.

RNA viruses, such as Deformed wing virus, are of particular interest due to their lack of host specificity and capacity to jump between hosts^3^,^4^,^5^,^6^. To enable this generalist infection strategy with little host specificity, Iflaviruses, such as DWV, exist as a cloud of variants known as a quasispecies^7^. The level of diversity or ‘cloud size’ within a viral quasispecies has been correlated with the host range size the virus^8^. Predicting virus emergence before epidemic spread allows mitigating action to be considered but is not always possible. As a general rule, the larger the reservoir species population size, the more viruses it can harbour, and as a consequence viruses with higher virulence arise more frequently^9^. The large population sizes and high densities of many insect populations provide a perfect environment for emerging viruses to arise and transmit freely. Social insects epitomise this, making up just 2% of all insect species, but more than half of the total insect biomass^10^. Transmission between hosts is facilitated by interactions between insect species, including predation and sharing of resources^3^.

It is becoming apparent that insects which interact with honey bees can act as viral reservoirs and infect honey bees via spillover events^3^,^11^,^12^. In addition, the introduction of invasive species through anthropogenic processes offers the opportunity for new hosts, with their own assortment of viromes, to be introduced to previously isolated populations such as those in the Hawaiian archipelago.

The predatory social wasp, *Vespula pensylvanica* is a common species native to the western half of temperate North America. In the Hawaiian archipelago, it was first recorded in Kauai in 1919, but was not recorded on Maui and the Big Island until 1978^13^ and has since flourished becoming a serious pest. *V. pensylvanica* is a general predator that feeds on a wide range of arthropods including the honey bee, *Apis mellifera*^13^. The ecological role of *V. pensylvanica* as an invasive species with widespread geographic distribution, abundant numbers in some areas and generalist feeding preferences including its use of floral resources^14^, make it important to investigate whether or not it could act as a reservoir for a new or emerging honey bee virus.

Here we report a novel RNA virus, genetically dissimilar to any other virus at the nucleotide level, detected in *V. pensylvanica*. We named the novel virus “Moku”, which means Island in Hawaiian.

## Results & Discussion

**Figure 1:**
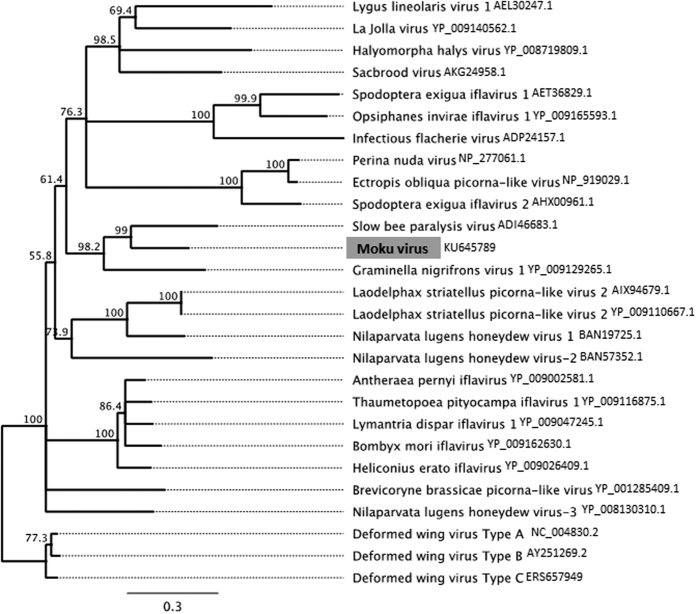
Neighbour joining tree using the amino acid sequences of a conserved region of the RdRP^29^. Values show the consensus support (%).

**Figure 2:**
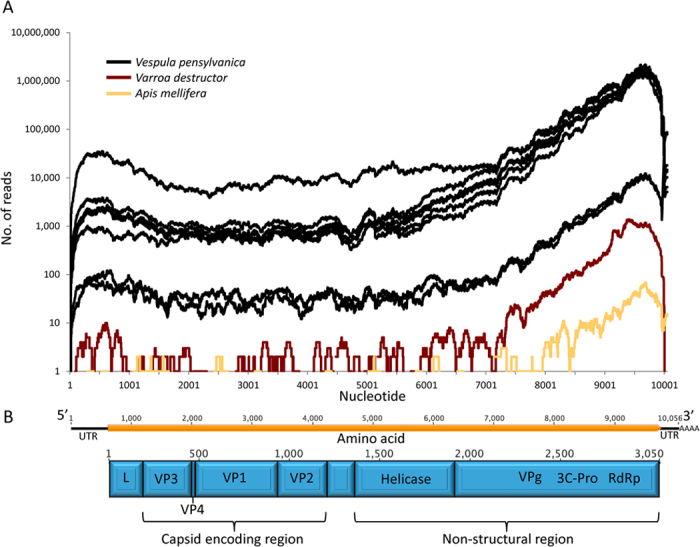
(**A**) Moku virus genome coverage from Illumina Hi-seq data for samples collected in Hawaii. *V. pensylvanica* are shown in black, Varroa in red and honey bees in yellow. Three different honey bee Illumina runs were pooled together for the honey bee data. (**B**) Organisation of the 10,056 nucleotides Moku virus genome (black line) coding for a 3050-aa polyprotein (orange box) and the predicted polyprotein coding regions are shown in blue.

A blastx search against the NCBI protein database revealed the polyprotein of MV shared 46% amino acid similarity to slow bee paralysis virus (SBPV) and phylogenetic analysis confirmed that Moku virus (MV) lies within the genus *Iflavirus* (Fig. 1). The genome is 10,056 nucleotides long (accession number KU645789) with a poly-A tail at the 3′ end and full genome coverage was observed in all the 8 wasp samples (Fig. 2A). The genome contains a 9153 nucleotide open reading frame encoding for 3,050 amino acids (Fig. 2B). In addition, partial genome-wide coverage was observed in *A. mellifera* and *Varroa destructor* collected on Big Island (Fig. 2A and Supplementary Fig. S1).

Further genome annotation was carried out by comparing the 3C protease sites with the previously annotated SBPV and DWV genomes^15^,^16^. The 3,050-aa polyprotein contained conserved domains typical of the iflaviruses, including capsid (cd00205, pfam08762, pfam00073), helicase (pfam00910), RNA dependent RNA polymerase (pfam 00680) and 3C protease (pfam00548) domains (Fig. 2B). These were arranged in the canonical *Iflavirus* genome structure; structural proteins in the N-terminal region and non-structural proteins in the C-terminal region (RNA helicase, protease, RdRp) (Fig. 2B). No conserved domain was recognised for the leader protein, the most variable region of the *Iflavirus* genome^15^. Assemblies of MV from each individual wasp using Vicuna^17^ showed that all wasp samples shared at least 98% nucleotide identity suggesting that MV is relatively invariant, at least in the wasps sampled. However, greater variation occurred between species. For example, when the RdRp, helicase and VP3 consensus regions of the MV genome from the honey bees and Varroa were aligned against the wasp MV genome, we observed roughly 2 nucleotide substitutions per 100 nucleotide base pairs for the RdRp region for both the honey bees and the Varroa (Supplementary Fig. S1A), and in Varroa one of these substitutions resulted in an amino acid change. Background genomic variation (likely due to mutations created during genome replication) can also be seen in the reads that represents the honey bee and Varroa MV quasispecies (Supplementary Fig. S2). This group of viruses exist as a cloud of variants^7^, therefore contigs generated by de novo assembly represent a consensus of the most dominant sequence. Although consensus sequences of MV were similar and belong to the same master variant, a cloud of mutants around the main consensus can be seen in individual reads (Supplementary Fig. S2).

Since we were unable to assemble a full-length MV genome from both the honey bee and Varroa samples, pairwise comparison between these genomes was not possible. Taken together, genomic variants of MV were observed in both the honey bee and Varroa samples. The amino acid identities between MV variants were highly conserved (Supplementary Fig. S1), therefore, these variants belong to the same master MV variant. MV is likely similar to DWV^7^, i.e. it exists as a quasispecies with variation around one or more master variant(s). Further screening and sequencing of MV in different hosts and geographic locations is likely to reveal further genetic variation within the virus quasispecies.

**Figure 3:**
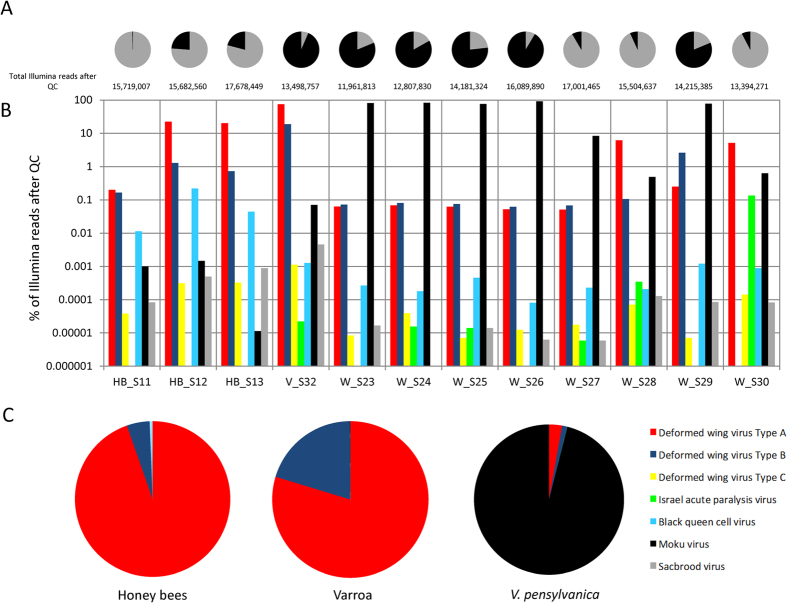
(**A**) Proportion of total Illumina Hi-Seq reads which were attributed to viruses by BLAST labelled with the total number of Illumina reads after QC. (**B**) Illumina Hi-Seq Virome for each sample (W = *V. pensylvanica*, V = Varroa, HB = honey bee) showing the number of top BLAST hits against a custom virus database. Note a logarithmic scale has been used to display the vast differences between viruses. C) Pie charts showing samples grouped per species showing the proportion of viral hits determined by BLAST.

Advancements in NGS technologies have enabled an exponential increase in RNA virus discovery^18^ yet only a few new honey bee viruses have been discovered since the pioneering work of Bailey & Ball^19^. Recent discoveries include a Macula-like virus in honey bees and Varroa^20^ and the replication of a plant virus (Tobacco ringspot Virus) in honeybees^21^. NGS technologies have shown that viral loads in insects can be high; for example, we have previously shown that DWV reads made up 46.3% of all Illumina reads in Varroa and 9.7% in honey bees^22^. Similarly, here we show that in *V. pensylvanica*, virus reads can make up to 91.5% of total Illumina reads (average of 54.6%) (Fig. 3A). Due to the quasispecies nature of the iflaviruses^7^, Moku virus was named after the location of its discovery rather than the host or disease symptoms (of which there is no particular phenotype recorded to date). It is likely that MV is able to replicate in several hosts, potentially with several master variants each of which differs in pathogenicity depending on the host. However, the high viral load (up to 99.87% of total virus reads in wasps, Fig. 3B,C) and full genome coverage (Fig. 2A) observed for MV in *V. pensylvanica* suggests it is likely to be MV’s native host. Use of “Moku” as a name also conforms to the International Committee on Taxonomy of Viruses (ICTV) preference not to use host species names to assign virus species^23^. Many insect RNA viruses discovered by NGS data^18^ do not result in overt symptoms, preventing the use of disease symptoms for virus classification.

Moku virus is most closely related to SBPV (Fig. 1). SBPV is highly virulent in honey bees but has only been found in the UK, Fiji and Western Samoa despite the numerous surveys across the world^16^. More recent evidence suggests that SBPV’s natural host is the bumble bee (*Bombus* spp.) rather than honey bees^24^. Similarly, wasps could act as the reservoir for Moku virus, which commonly circulates in the vespine host, but with the potential to be highly virulent in honey bees, as with SBPV. This is worthy of regular monitoring as invasive species such as *V. pensylvanica* in Hawaii and *Vespa velutina* (Asian hornet) in Europe could act as a new transmission route and source of emerging viruses in honey bees.

Detection of MV in Varroa is of concern, as Varroa is known to facilitate the spread and amplification of some RNA viruses^1^. Our data suggests a possible transmission route of MV from *Vespula pensylvanica* to honey bees (or vice versa as *V. pensylvanica* is known to predate on honey bees) and then onto Varroa. Once in Varroa, transmission at epidemic proportions within honey bee populations is a possible outcome^1^. However, DWV still dominates the virome of honey bees with only low levels of MV detected. This suggests that currently, honey bees and Varroa are not the likely origin of MV, however, negative strand RT-PCR tests^4^ must now be used to reveal replication efficiency of MV in different hosts and tissue types. As well as preying on arthropods, *V. pensylvanica* supplements its diet by feeding on floral nectar^14^, a shared resource, which could be a possible route of transmission of MV from wasp to honey bee. However, the mechanism of transmission is yet to be determined; further screening for MV is required to determine the full host range and indeed, epidemiology of this virus.

The dominance of the DWV type A master-variant in honey bees and Varroa is the result of the arrival of Varroa on Big Island, which facilitated the spread of this variant^1^. Reads identified as Moku virus, DWV type C. Sacbrood virus and Black queen cell virus were only present at low levels compared to the high number of DWV type A reads, and to a lesser extent DWV type B reads (Fig. 3). Only a very small number of reads (0-200 range) were attributed to DWV type C^7^.

A recent study demonstrated *in vitro* that DWV can cause premature death of adult honey bees^25^. The honey bee harbours a lethal cocktail of RNA viruses, which dependent on circumstance (environmental conditions, anthropogenic stressors or the introduction of a new vector) can result in the most severe of outcomes. In addition, insects often found associated with honey bees also carry highly virulent RNA viruses. This is evident by the presence of Israeli acute paralysis virus (IAPV) in *V. pensylvanica* (Fig. 3). In one wasp sample, IAPV reads make up 1.7% of the total virus reads sequenced; despite no IAPV reads being detected in the honey bee samples from the same location on the same day. This suggests that IAPV is replicating in the vespine host. ‘Honey bee’ RNA viruses are generalists, capable of infecting a variety of insect hosts^3^,^4^ and they can be readily transmitted between hymenoptera insects such as bees and now wasps.

The pathogenicity of Moku virus in wasps and honey bees remains unknown. The high viral load of MV in wasps suggests that *V. pensylvanica* is its natural host. Interestingly, two of the wasp samples (W_S28 and W_S30) contained several orders of magnitude less MV reads than the other wasp samples (Fig. 3). These two samples were instead dominated by DWV (type A & B), suggesting that there is a possible competitive interaction between MV and DWV, plausibly for sites of replication. Therefore, it is possible that the relatively high viral loads of DWV in honey bees and Varroa effectively exclude Moku virus from replicating to higher levels. Competitive exclusion has previously been suggested between iflaviruses where a persistent DWV infection *in vitro* was suggested to restrict the replication of a related virus^26^ as well as *in vivo* where one variant of DWV prevented superinfection by another^22^. The detection of Moku virus in wasps, Varroa and honey bees suggests that cross-species transmission of RNA viruses is a threat to pollinator health worldwide. This is further supported by the recent discovery of a plant virus replicating in honey bees, demonstrating the host range of RNA viruses can even cross kingdoms^21^.

## Methods

RNA was extracted from eight asymptomatic *V. pensylvanica* individuals collected from managed honey bee apiaries on Big Island, Hawaii in 2012. Bees were sampled from the frames inside the hive, so will likely be mostly nurses with some foragers and newly emerged bees. Samples W_S23-27 and HB_S11-12 were collected from the North of Big Island, and samples HB_S13, V_S32 and W_S28-30 were from the East. 30 honey bees were pooled for RNA extraction. The Varroa samples were a pool of 10 mites taken from drone brood. cDNA libraries were prepared using oligo dT priming followed by Illumina 2 × 100 bp Hiseq sequencing.

QC was done using FastQC (http://www.bioinformatics.babraham.ac.uk/projects/fastqc/) to confirm the quality of the raw read data. An in-house contamination-screening pipeline called Kontaminant (http://www.tgac.ac.uk/kontaminant/) was used to check for any contamination in the raw reads. The wasp libraries showed less than 5% of host mRNA. Even with very low host contamination, kmer filtering was performed to remove any host RNA. There was no viral mapping/filtering done, so we carried out a metagenomic study to assemble all the non-host RNA.

From a total of 8 wasp individuals, around 116 million reads (115, 842, 147 total reads before filtering) were assembled together in a single assembly run using MetaCortex (Unpublished, developed by Richard Leggett in TGAC). MetaCortex is a recently developed variant of Cortex^27^,^28^ based on de Bruijn graphs, which are constructed by dividing reads into smaller, overlapping sequences called kmers. Contigs were aligned (blastx) against a refseq protein database (NCBI) to identify putative viruses.

One contig in particular was translated within Geneious (Biomatters) and aligned with other *Iflavirus* amino acid sequences obtained from Genbank. The alignment was carried out using the Muscle aligner with 8 iterations. The phylogenetic tree was built by the Geneious tree builder using a neighbour joining method and the Jukes-Cantor genetic distance model based on the conserved RdRP region of picorna-like viruses^29^. Finally, Geneious was used to map reads against the putative virus contig and Vicuna^17^ was used to assemble reads from each individual separately using a pipeline adapted from assembling DWV^7^.

Individual reads were aligned against the novel Moku virus genome to create coverage plots for each Illumina sample (Fig. 2A). From these reads a consensus of the RdRp region was obtained for MV in Varroa and honey bees by keeping bases that match at least 90% of the sequences. The Moku virus genome was annotated based on an amino acid alignment with the SBPV and DWV genomes^15^,^16^. Regions were identified by protease sites based on the DWV and SBPV genomes and homologous protein domains identified by BLAST. Reads from Varroa and honey bees were pulled out and made into a consensus and aligned with the MV genome from the wasps to confirm that they were indeed MV (Supplementary Figs S1 and S2).

The insect viromes (Fig. 3B) were created by aligning individual Illumina reads using BLAST against a custom virus database which included the Moku virus genome, Slow bee paralysis virus, and all three variants of DWV^7^. The top hits were counted for each viral species. BLAST hits of individual reads that did not cover the whole read were excluded from the analysis.

## Additional Information

**How to cite this article**: Mordecai, G. J. *et al*. Moku virus; a new *Iflavirus* found in wasps, honey bees and Varroa. *Sci. Rep.* **6**, 34983; doi: 10.1038/srep34983 (2016).

## Supplementary Material

Supplementary Information
