# Supplementary material for: Moku virus; a new Iflavirus found in wasps, honey bees and Varroa
Source: Sci Rep. 2016 Oct 7;6:34983. doi: 10.1038/srep34983 (PMC5054524; doi:10.1038/srep34983)
Supplement: Supplementary Information [file srep34983-s1.pdf]

Supplementary Information for:

Moku virus; a new *Iflavirus* found in wasps, honey bees and Varroa.

Gideon J Mordecai, Laura E Brettell, Purnima Pachori, Ethel M. Villalobos, Stephen J Martin, Ian M Jones, and Declan C Schroeder

Content:

1) Supplementary Figure S1

2) Supplementary Figure S2

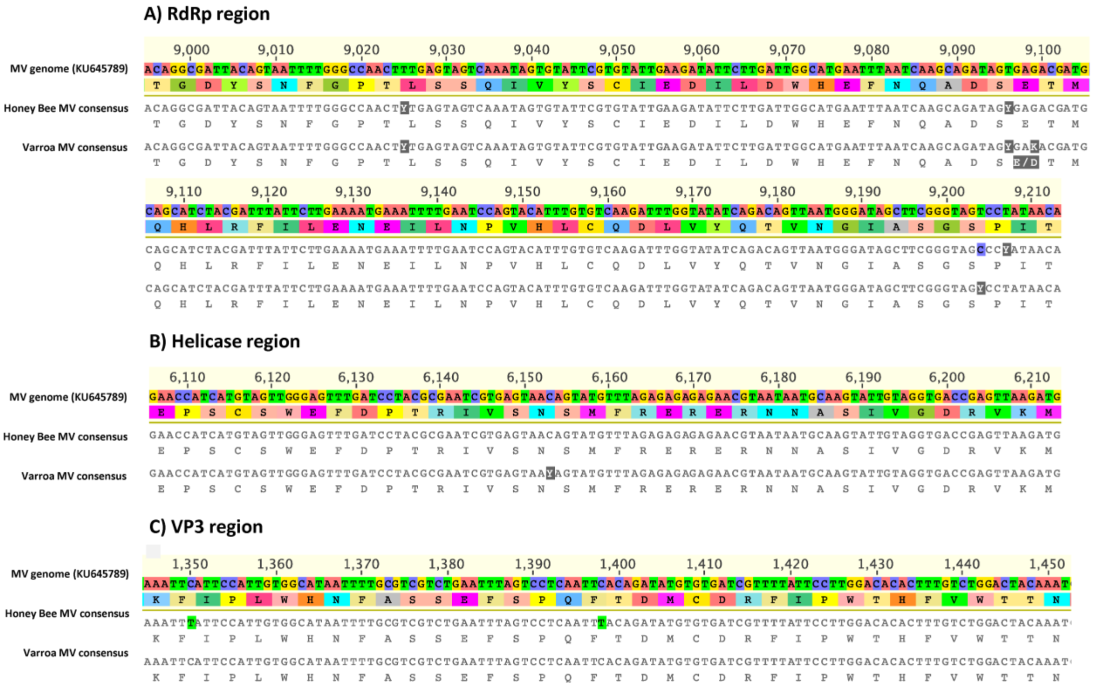

Supplementary Figure S1. Multiple sequence alignment of three MV genomic regions of the MV genome showing translated consensus sequence from honey bees and Varroa aligned against the wasp MV genome (KU645789). Substitutions against the MV genome are highlighted.

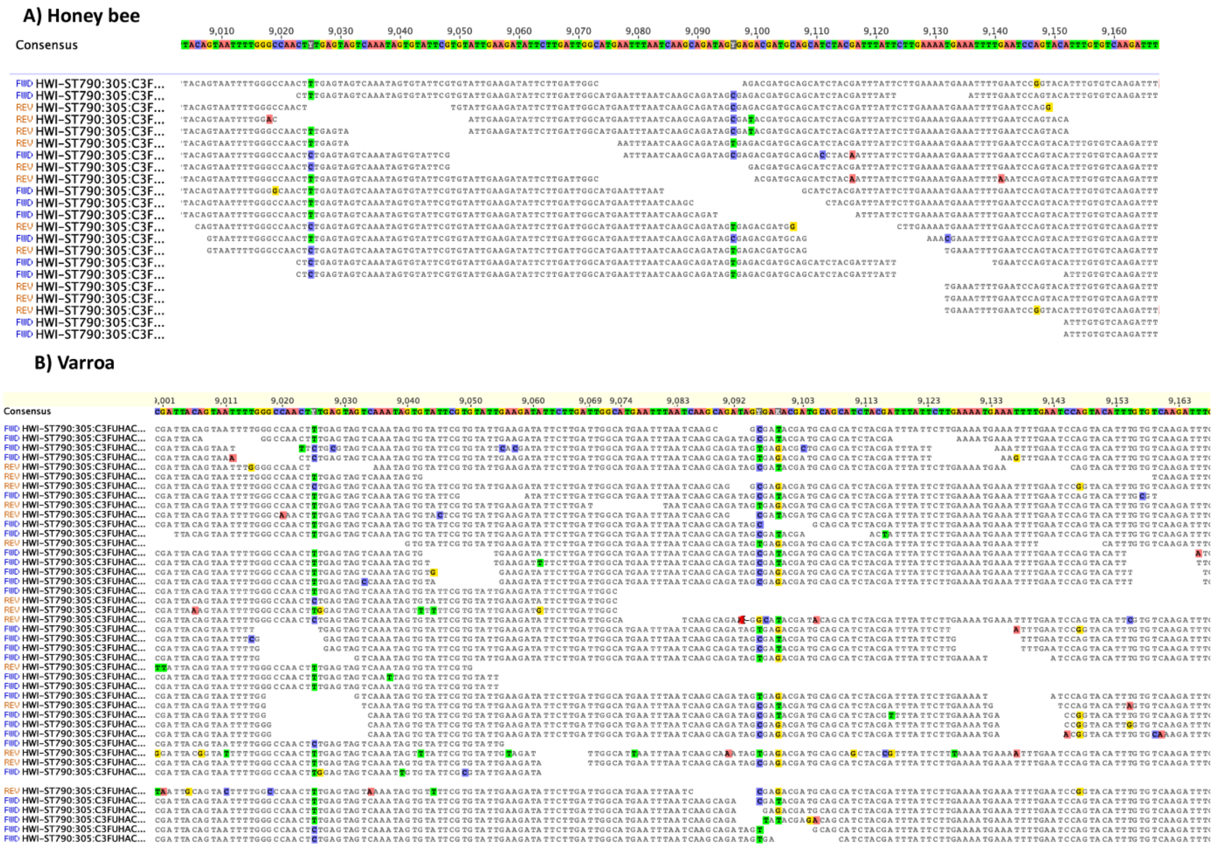

Supplementary Figure S2. A screenshot of the Illumina reads mapped to the RdRP consensus region of MV genomes in Honey bee (A) and Varroa (B) as described in Supplementary Figure S1. Substitutions against the MV genome are highlighted
